# Effectiveness of bivalent mRNA booster vaccination against SARS-CoV-2 Omicron infection, the Netherlands, September to December 2022

**DOI:** 10.2807/1560-7917.ES.2023.28.7.2300087

**Published:** 2023-02-16

**Authors:** Anne J Huiberts, Brechje de Gier, Christina E Hoeve, Hester E de Melker, Susan JM Hahné, Gerco den Hartog, Janneke HHM van de Wijgert, Susan van den Hof, Mirjam J Knol

**Affiliations:** 1 Centre for Infectious Disease Control, National Institute for Public Health and Environment (RIVM), Bilthoven, the Netherlands; 2 Radboud Centre for Infectious Diseases, Radboudumc, Nijmegen, the Netherlands; 3 Julius Centre for Health Sciences and Primary Care, University Medical Centre Utrecht (UMCU), Utrecht, the Netherlands

**Keywords:** COVID-19, vaccine effectiveness, SARS-CoV-2

## Abstract

We used data of 32,542 prospective cohort study participants who previously received primary and one or two monovalent booster COVID-19 vaccinations. Between 26 September and 19 December 2022, relative effectiveness of bivalent original/Omicron BA.1 vaccination against self-reported Omicron SARS-CoV-2 infection was 31% in 18–59-year-olds and 14% in 60–85-year-olds. Protection of Omicron infection was higher than of bivalent vaccination without prior infection. Although bivalent booster vaccination increases protection against COVID-19 hospitalisations, we found limited added benefit in preventing SARS-CoV-2 infection.

The severe acute respiratory syndrome coronavirus 2 (SARS-CoV-2) Omicron (Phylogenetic Assignment of Named Global Outbreak lineage (Pango) lineage B.1.1.529) variant has been dominant in Europe since January 2022, causing large waves of infections because of high transmissibility and escape from vaccine- and infection-induced immunity [1]. Bivalent mRNA vaccines targeting the Omicron BA.1 subvariant and the original Wuhan strain of SARS-CoV-2 [2] have been available as booster vaccination for all individuals 12 years and older in the Netherlands since 19 September 2022. At that time, the Omicron BA.5 subvariant and not the Omicron BA.1 subvariant was the dominant Omicron subvariant in the Netherlands [3]. Individuals 60 years and older, medical risk groups and healthcare workers were invited by personal letters. 

We present estimates of the relative effectiveness of bivalent Omicron BA.1-targeted vaccination against self-reported SARS-CoV-2 Omicron infection between 26 September and 19 December 2022 among adults who had previously received primary vaccination and one or two monovalent booster vaccinations.

## Study population

We used data from 32,542 participants of an ongoing prospective cohort study (VASCO) among community-dwelling Dutch adults aged 18–85 years who are followed with 3-monthly questionnaires and 6-monthly serum samples [4,5]. We only included participants who had received primary vaccination and one or two monovalent booster vaccinations before the start of the bivalent booster programme (19 September 2022). Follow-up started on 26 September 2022 (1 week after the start of the bivalent booster vaccination programme), or 3 months after the last monovalent vaccination or last prior infection (occurring before 26 September 2022), whichever came last. This is in line with vaccination policy, where individuals are eligible for a bivalent vaccine 3 months after vaccination or infection. Follow-up ended on 19 December 2022, at the date of first positive SARS-CoV-2 test or at the date of last completed follow-up questionnaire, whichever came first. 

We included 12,988 participants aged 18–59 years who had previously received a primary vaccination series and one monovalent booster vaccination. We further included 19,554 participants aged 60–85 years who had previously received a primary vaccination series and one (n = 8,963) or two (n = 10,591) monovalent booster vaccinations. In total, 5,504 (42.4%) 18–59-year-olds and 11,900 (60.9%) 60–85-year-olds received a bivalent vaccine after 19 September 2022 (Table). Prior SARS-CoV-2 infection, based on self-report or presence of anti-nucleoprotein antibodies [4], was present in 9,605 (74.0%) of 18–59-year-olds and 10,898 (55.7%) of 60–85-year-olds (Table). Participants who received the bivalent booster vaccine were older (median age: 51 vs 48 years in 18–59-year-olds) and more often had a medical risk condition (26.5% vs 18.0% in 18–59-year-olds; 41.9% vs 38.2% in 60–85-year-olds) than participants who did not receive a bivalent booster. Among 60–85-year-olds, the bivalent booster vaccine recipients had more frequently received two prior monovalent booster vaccinations than the non-recipients (58.2% vs 47.9%).

**Table:** Characteristics of participants included in the analysis of SARS-CoV-2 bivalent vaccine effectiveness, the Netherlands, September–December 2022 (n = 32,542)

	18–59 years							60–85 years						
	Overall		Bivalent booster vaccination		No bivalent booster vaccination^a^		p value	Overall		Bivalent booster vaccination		No bivalent booster vaccination^a^		p value
	n	%	n	%	n	%		n	%	n	%	n	%	
All participants	12,988	100	5,504	100	7,484	100	NA	19,554	100	11,900	100	7,654	100	NA
Median age in years (IQR)	49 (15)		51 (12)		48 (16)		<0.001	66 (6)		66 (6)		65 (6)		0.054
Sex														
Female	9,497	73.1	4,115	74.8	5,382	71.9	<0.001	10,797	55.2	6,584	55.3	4,213	55.0	0.427
Male	3,484	26.8	1,388	25.2	2,096	28.0		8,756	44.8	5,316	44.7	3,440	44.9	
Other	7	0.1	1	0	6	0.1		1	0	0	0	1	0	
Prior infection^b^														
No prior infection	3,383	26.0	1,497	27.2	1,886	25.2	0.027	8,656	44.3	5,431	45.6	3,225	42.1	<0.001
Prior pre-Omicron infection	1,331	10.2	569	10.3	762	10.2		2,105	10.8	1,246	10.5	859	11.2	
Prior Omicron infection	8,274	63.7	3,438	62.5	4,836	64.6		8,793	45.0	5,223	43.9	3,570	46.6	
Medical risk condition^c^, yes	2,803	21.6	1,457	26.5	1,346	18.0	<0.001	7,913	40.5	4,989	41.9	2,924	38.2	<0.001
Cardiovascular disease	1,038	8.0	557	10.1	481	6.4		5,099	26.1	3,242	27.2	1,857	24.3	
Lung disease or asthma	1,008	7.8	554	10.1	454	6.1		1,517	7.8	1,001	8.4	516	6.7	
Diabetes mellitus	289	2.2	166	3.0	123	1.6		1,298	6.6	801	6.7	497	6.5	
Immune deficiency	226	1.7	105	1.9	121	1.6		308	1.6	190	1.6	118	1.5	
Monovalent vaccination status before study period^d^														
Booster 1	12,988	100	5,504	100	7,484	100	NA	8,963	45.8	4,976	41.8	3,987	52.1	<0.001
Booster 2	NA							10,591	54.2	6,924	58.2	3,667	47.9	
Education level^e^														
High	8,266	63.6	3,674	66.8	4,592	61.4	<0.001	10,334	52.8	6,511	54.7	3,823	49.9	<0.001
Intermediate	3,901	30.0	1,527	27.7	2,374	31.7		5,253	26.9	3,128	26.3	2,125	27.8	
Low	783	6.0	293	5.3	490	6.5		3,818	19.5	2,174	18.3	1,644	21.5	
Other	38	0.3	10	0.2	28	0.4		149	0.8	87	0.7	62	0.8	
Bivalent vaccine product														
Spikevax	NA		2,689	48.9	NA		NA	NA		9,431	79.3	NA		NA
Comirnaty	NA		2,687	48.8	NA			NA		1,774	14.9	NA		
Unknown	NA		128	2.3	NA			NA		695	5.8	NA		
Time between bivalent vaccine and end of follow-up														
Median (days)	NA		33		NA		NA	NA		39		NA		NA
Test intention^f^														
High	10,368	79.8	4,748	86.3	5,620	75.1	<0.001	16,306	83.4	10,401	87.4	5,905	77.1	<0.001
Middle/low	2,620	20.2	756	13.7	1,864	24.9		3,248	16.6	1,499	12.6	1,749	22.9	

COVID-19: coronavirus disease; IQR: interquartile range; NA: not applicable; SARS-CoV-2: severe acute respiratory syndrome coronavirus 2.

^a^ Participants could only receive bivalent booster vaccination during the study period; no other COVID-19 vaccinations were provided.

^b^ Prior infection at least 3 months before start of follow-up; pre-Omicron infection was defined as positive test date before 20 December 2021; Omicron infection was defined as positive test date after 9 January 2022; participants with prior infection in transition period from Delta to Omicron (20 December 2021–9 January 2022) were excluded; prior infection was based on self-reported test-confirmed infections or the presence of anti-nucleoprotein antibodies before the start of the study period. Date of prior infection based on anti-nucleoprotein antibodies (no corresponding infection reported) was imputed as mid-date between two blood samples, where the first was negative and the second was positive for anti-nucleoprotein antibodies, or where the second had at least a four‐fold increase in anti-nucleoprotein antibody concentration compared with the first. When the first serum sample of a participant was positive for anti-nucleoprotein antibodies, but no prior infection was reported, an infection date was imputed as the mid‐date between the baseline questionnaire and sample receipt. Serological analyses were done as previously described [4]. Of 31,448 participants (97%), at least one anti-nucleoprotein antibody result was available, with a median time of 104 days between last blood sample and start follow-up. Blood sample data were available until 16 September 2022.

^c^ Medical risk condition: one or more of the following conditions: diabetes mellitus, lung disease or asthma, asplenia, cardiovascular disease, immune deficiency, cancer (currently untreated, currently treated, untreated), liver disease, neurological disease, renal disease, organ or bone marrow transplantation.

^d^ Participants who received a third dose before the start of the general public booster campaign (18 November 2021) were excluded. Persons 60 years or older and only a very high-risk group younger than 60 years were eligible for second booster vaccination; therefore, participants younger than 60 years with a second booster vaccination were excluded.

^e^ Educational level was classified as low (no education or primary education), intermediate (secondary school or vocational training) or high (bachelor’s degree, university).

^f^ Information on test intention was obtained from the questionnaires. Participants were classified as high when in all questionnaires during the study period they answered “almost always” or “always” to the question whether they (would) test when having symptoms.

## Incidence of SARS-CoV-2 infection

**Figure 1:**
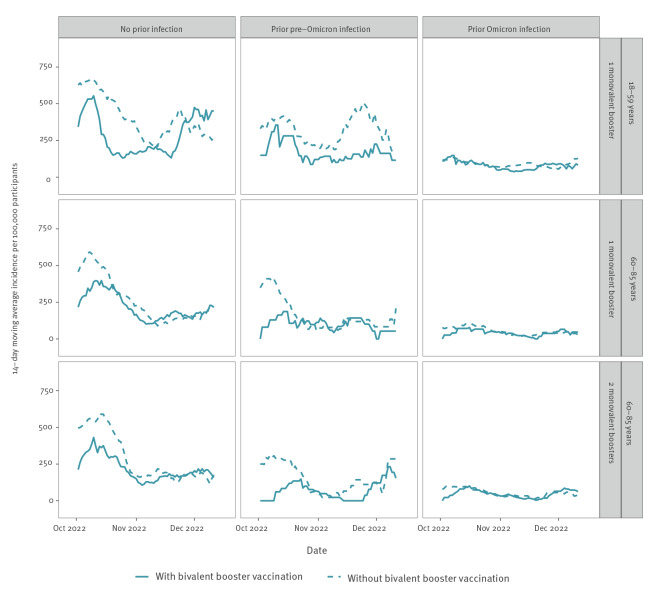
14-day moving average of number of SARS-CoV-2 infections reported per 100,000 participants by age group, prior infection status and vaccination status, the Netherlands, 26 September–19 December 2022 (n = 32,542)

During the study period, 3,005 SARS-CoV-2 infections, based on a positive SARS-CoV-2 PCR or (self-administered) antigen test, were reported by the participants. The reported incidence in September and October 2022 was high (Figure 1), consistent with national data from syndromic and wastewater surveillance [6,7]. The incidence was highest among participants without any prior infection, lower among participants with a prior pre-Omicron infection, and lowest among participants with a prior Omicron infection. During most of the study period, the incidence was lower among participants who did receive than among those who did not receive a bivalent booster vaccine. However, it is important to note that the number of participants with a bivalent vaccine was small at the beginning of the study period and thus the incidence in these participants was based on a small number of infections. We provide further details on the number of participants and infections per vaccination status in Supplementary Figure S1.

SARS-CoV-2: severe acute respiratory syndrome coronavirus 2.

## Relative vaccine effectiveness

To estimate effectiveness of bivalent vaccination relative to receiving the primary vaccination series and one or two monovalent booster vaccinations, we used Cox proportional hazard models with calendar time as underlying time scale and bivalent vaccination as time-varying exposure. Estimates were adjusted for age group, sex, education level and presence of a medical risk condition. We present stratified estimates by infection history and an overall estimate additionally adjusted for infection history. The 7 person-days after bivalent vaccine administration were excluded. All analyses were done using R version 4.2.2 (R Foundation, Vienna, Austria) and packages Epi and survival.

**Figure 2:**
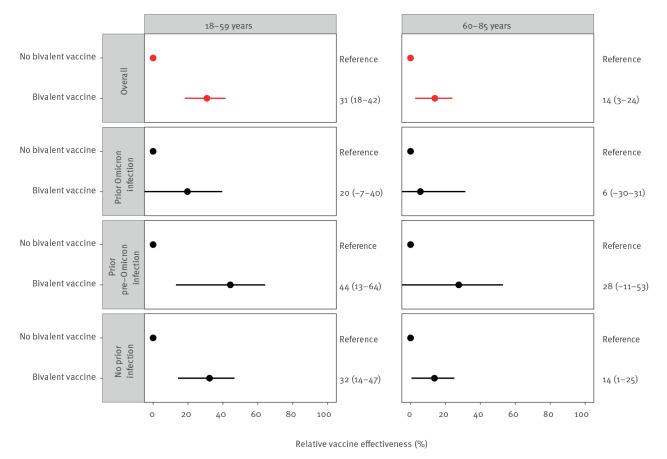
Relative vaccine effectiveness^a^ and 95% confidence interval of bivalent COVID-19 vaccine overall and stratified by infection history and by age group, the Netherlands, 26 September 2022–19 December 2022 (n = 32,542)

Among 18–59-year-olds who received primary vaccination and one monovalent booster, the overall relative effectiveness of bivalent vaccination against infection was 31% (95% confidence interval (CI): 18 to 42). Among participants with prior Omicron infection the relative effectiveness of a bivalent booster appeared lower (20%; 95% CI: −7 to 40) than among participants with no prior infection (32%; 95% CI: 14 to 47) or prior pre-Omicron infection (44%; 95% CI: 13 to 64), although confidence intervals largely overlapped (Figure 2). Among 60–85-year-olds who received primary vaccination and one or two monovalent booster vaccinations, overall relative effectiveness was 14% (95% CI: 3 to 24). Among participants with prior Omicron infection this was 6% (95% CI: −30 to 31). We provide number of infections, person time and estimates with confidence intervals per age group in Supplementary Table S1.

COVID-19: coronavirus disease.

^a^ Adjusted for age group (18–39, 40–59, 60–69, 70–85), sex, education level and presence of a medical risk condition; overall estimates were additionally adjusted for infection history.

**Figure 3:**
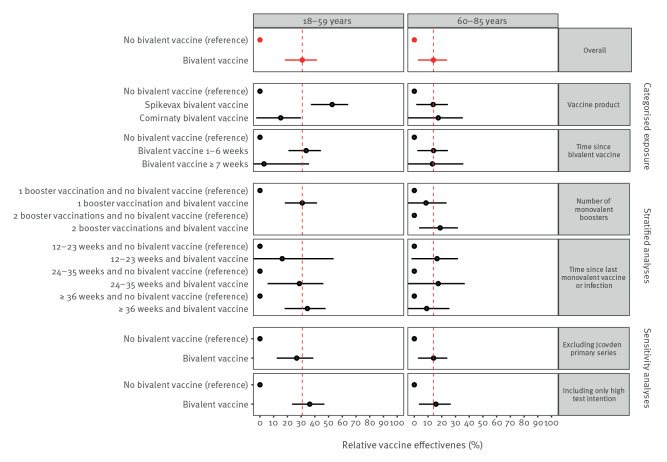
Stratified and sensitivity analyses^a^ for relative vaccine effectiveness of bivalent COVID-19 vaccination by age group, the Netherlands, 26 September 2022–19 December 2022 (n = 32,452)

Estimates among 60–85-year-olds were similar to the main estimate across different stratified analyses and sensitivity analyses (Figure 3). Among 18–59-year-olds, stratification by bivalent vaccine product showed higher relative effectiveness of Spikevax (mRNA-1273, Moderna, Cambridge, United States (US)) than of Comirnaty (BNT162b2 mRNA, BioNtech/Pfizer, Mainz, Germany/New York, US) bivalent vaccine; of note, Spikevax was only given to individuals 45 years and older and therefore the median age in Spikevax recipients was higher than in Comirnaty recipients (54 vs 43 years) (Figure 3).

COVID-19: coronavirus disease.

^a^ Adjusted for age group (18–39, 40–59, 60–69, 70–85), sex, education level, presence of a medical risk condition, and infection history. Categorised exposure = exposure categorised by (i) vaccine product (n = 31,719) and (ii) time since bivalent vaccination (n = 32,542); Stratified analyses = analyses stratified by (i) number of monovalent booster doses before start of follow-up (n = 32,542) and (ii) time since last monovalent booster at start follow-up (n = 32,542); Sensitivity analyses = analyses performed (i) excluding all individuals with a Jcovden (Ad26.COV2-S (recombinant), Janssen-Cilag International NV, Beerse, Belgium) primary series (n = 29,577)) and (ii) in only persons with a high intention to test during follow-up (n = 26,674).

In participants aged 18–59 years, compared with those without bivalent vaccination and without prior infection, relative effectiveness of bivalent vaccination among participants without prior infection (37%; 95% CI: 21 to 50) was similar to relative protection from a prior pre-Omicron infection and no bivalent vaccination (34%; 95% CI: 21 to 44), while relative protection from a prior Omicron infection with or without bivalent vaccination was substantially higher (80–83%). Similarly, participants aged 60–85 years showed higher relative protection from prior Omicron infection with (82%; 95% CI: 76 to 86) or without bivalent vaccination (82%; 95% CI: 79 to 85) than from bivalent vaccination (14%; 95% CI: 1 to 25) or prior pre-Omicron infection (43%; 95% CI: 32 to 52). We provide estimates with confidence intervals in Supplementary Table S2.

## Discussion

We found that Omicron BA.1-targeted bivalent vaccination gave an overall relative vaccine effectiveness against Omicron SARS-CoV-2 infection of 31% in 18–59-year-olds and 14% in 60–85-year-olds who had previously received the primary vaccination series and at least one booster vaccination, adjusted for infection history.

Estimates of (relative) effectiveness of bivalent vaccination against infection are scarce. A recent study from the US reported slightly higher estimates against infection by the BA.4/BA.5-targeted bivalent vaccine (respectively 46%, 38% and 36% 6–7 months after last monovalent dose in individuals aged 18–49 years, 50–64 and ≥ 65 years) [8]. However, these estimates were not stratified by or adjusted for infection history. A preprint publication from the Nordic countries reported a relative effectiveness against hospitalisation of 75% for the BA.1-targeted bivalent vaccine in individuals aged ≥ 50 years [9]. Dutch surveillance data reported a relative risk reduction of 45% in 40–59-year-olds and of 58% after BA.1-targeted bivalent vaccination in individuals aged ≥ 60 years with at least one prior monovalent vaccination [10].

Our data showed that prior Omicron infection provided higher protection than bivalent vaccination among persons without prior infection, even though the time since prior Omicron infection was longer than the time since bivalent vaccination. This is consistent with a recent preprint publication estimating higher and longer protection after a breakthrough infection compared with booster vaccination [11]. In general, a combination of vaccination and infection, i.e. hybrid immunity, has been shown to provide better protection against infection than vaccination alone [12,13]. We did, however, find that prior pre-Omicron infection had a similar effect as bivalent vaccination without prior infection, probably because the time since pre-Omicron infection was substantially longer than the time since bivalent vaccination.

The VASCO cohort participants were given SARS-CoV-2 self-administered antigen tests free of charge, and we were not dependent on the SARS-CoV-2 testing infrastructure. In addition, serological data allowed us to detect prior untested (asymptomatic) infections. Confidence intervals overlap for the stratified analyses according to infection history, which makes it difficult to conclude there are real differences. Estimates can be confounded through differences in factors between participants who did and did not receive a bivalent booster vaccine, including test frequency and differences in exposure through behaviour. Participants who received a bivalent booster vaccine had a slightly higher intention to test, but restricting the analysis to participants with high test intention did not change our estimates. Since we investigated only participants who already received monovalent booster vaccination (so no unvaccinated individuals) and COVID-19 measures were limited during the study period, differences in SARS-CoV-2 exposure between bivalent vaccine recipients and non-recipients are likely to be limited. We will probably have missed some infections during the study period because of self-reporting, but we think this will have been comparable between persons with and without the bivalent vaccine. 

## Conclusion

The bivalent booster vaccination campaign has shown benefit in reducing COVID-19 hospitalisations, which is especially important for those at increased risk, including elderly people and those with a medical risk condition. However, we found limited added protection of bivalent vaccination in preventing SARS-CoV-2 Omicron infection among persons who received primary vaccination and one or two monovalent booster vaccinations. Especially in persons with prior Omicron infection, the added benefit seems small. 

## Supplementary Data

373000 Supplement
