# Supplementary material for: Effectiveness of bivalent mRNA booster vaccination against SARS-CoV-2 Omicron infection, the Netherlands, September to December 2022
Source: Euro Surveill. 2023 Feb 16;28(7):2300087. doi: 10.2807/1560-7917.ES.2023.28.7.2300087 (PMC9936593; doi:10.2807/1560-7917.ES.2023.28.7.2300087)
Supplement: Supplement [file 23-00087_KNOL_Supplement.pdf]

Supplementary file - Effectiveness of bivalent mRNA booster vaccination against SARS-CoV-2 Omicron infection, the Netherlands, September to December 2022

This supplementary material is hosted by *Eurosurveillance* as supporting information alongside the article *Effectiveness of bivalent mRNA booster vaccination against SARS-CoV-2 Omicron infection, the Netherlands, September to December 2022*, on behalf of the authors, who remain responsible for the accuracy and appropriateness of the content. The same standards for ethics, copyright, attributions and permissions as for the article apply. Supplements are not edited by *Eurosurveillance* and the journal is not responsible for the maintenance of any links or email addresses provided therein.

Figure S1. Number of participants and reported SARS-CoV-2 infections per vaccination status over time stratified by infection history and age group from 26 September 2022 to 19 December 2022

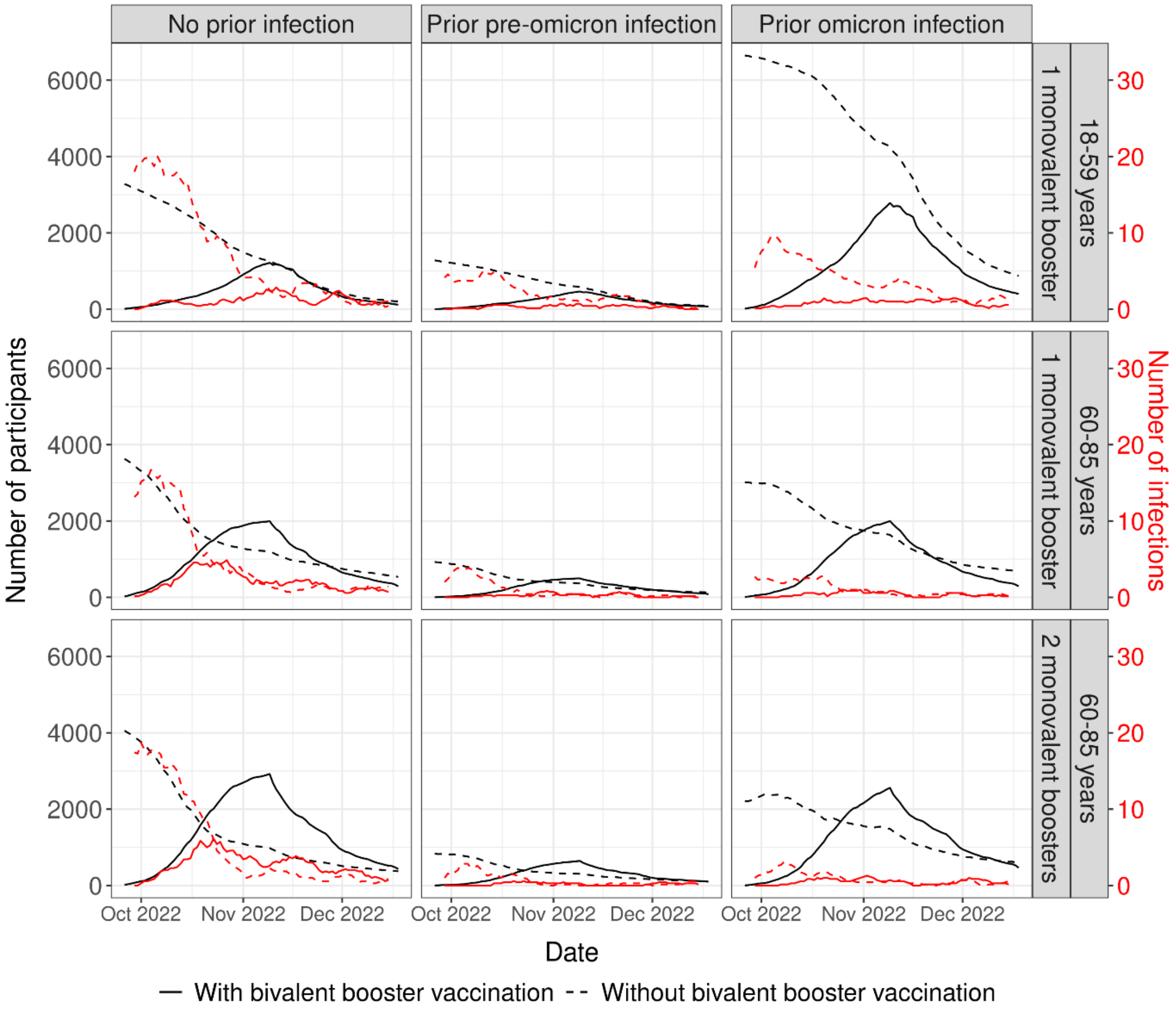

Table S1. Incidence rate and relative vaccine effectiveness of bivalent vaccine stratified by infection history and age group from 26 September 2022 to 19 December 2022

	Person-weeks	Number of infections	Rate (per 1,000 weeks)	Adjusted[a] relative vaccine effectiveness (95% CI)
**18-59 years**				
Overall				
No bivalent vaccine	73,298	1,112	15.2	Reference
Bivalent vaccine	22,543	190	8.4	31 (18-42)
No prior infection				
No bivalent vaccine	17,690	622	35.2	Reference
Bivalent vaccine	5,882	98	16.7	32 (14-47)
Prior pre-omicron infection				
No bivalent vaccine	7,433	166	22.3	Reference
Bivalent vaccine	2,419	26	10.7	44 (13-64)
Prior omicron infection				
No bivalent vaccine	48,174	324	6.7	Reference
Bivalent vaccine	14,242	66	4.6	20 (-7-40)
**60-85 years**				
Overall				
No bivalent vaccine	81,484	1,219	15.0	Reference
Bivalent vaccine	58,799	484	8.2	14 (3-24)
No prior infection				
No bivalent vaccine	34,677	917	26.4	Reference
Bivalent vaccine	28,045	381	13.6	14 (1-25)
Prior pre-omicron infection				
No bivalent vaccine	9,385	138	14.7	Reference
Bivalent vaccine	6,295	35	5.6	28 (-11-53)
Prior omicron infection				
No bivalent vaccine	37,422	164	4.4	Reference
Bivalent vaccine	24,458	68	2.8	6 (-30-31)

[a] Adjusted for age group (18-39, 40-59, 60-69, 70-85), sex, education level and chronic condition. Overall estimates were additionally adjusted for infection history.

Table S2. Effectiveness[a] of bivalent vaccination and prior infection status stratified by age group from 26 September 2022 to 19 December 2022

	No prior infection	Prior pre-omicron infection	Prior omicron infection
**18-59 years**			
No bivalent vaccination	Reference	34 (21-44)	80 (77-83)
Bivalent vaccination	37 (21-50)	60 (41-73)	83 (78-87)
**60-85 years**			
No bivalent vaccination	Reference	43 (32-52)	82 (79-85)
Bivalent vaccination	14 (1-25)	63 (48-74)	82 (76-86)

[a] Adjusted for age group (18-39, 40-59, 60-69, 70-85), sex, education level and chronic condition

Table S3. Incidence rate and relative vaccine effectiveness of bivalent vaccine in participants who (almost) always test in case of COVID-19 symptoms stratified by infection history and age group from 26 September 2022 to 19 December 2022

	**Person-weeks**	**Number of infections**	**Rate (per 1,000 weeks)**	**Adjusted[a] relative vaccine effectiveness (95% CI)**
**18-59 years**				
Overall				
No bivalent vaccine	55,397	934	16.9	Reference
Bivalent vaccine	18,989	163	8.6	36 (23-47)
No prior infection				
No bivalent vaccine	14,097	543	38.5	Reference
Bivalent vaccine	5,029	83	16.5	40 (22-53)
Prior pre-omicron infection				
No bivalent vaccine	5,700	132	23.2	Reference
Bivalent vaccine	2,004	22	11.0	47 (14-67)
Prior omicron infection				
No bivalent vaccine	35,600	259	7.3	Reference
Bivalent vaccine	11,956	58	4.9	23 (-5-44)
**60-85 years**				
Overall				
No bivalent vaccine	62,267	1,022	16.4	Reference
Bivalent vaccine	49,388	418	8.5	16 (3-27)
No prior infection				
No bivalent vaccine	26,135	763	29.2	Reference
Bivalent vaccine	23,615	330	14.0	15 (1-28)
Prior pre-omicron infection				
No bivalent vaccine	7,093	118	16.6	Reference
Bivalent vaccine	5,106	32	6.3	25 (-19-53)
Prior omicron infection				
No bivalent vaccine	29,039	141	4.9	Reference
Bivalent vaccine	20,667	56	2.7	11 (-26-38)

[a] Adjusted for age group (18-39, 40-59, 60-69, 70-85), sex, education level and chronic condition. Overall estimates were additionally adjusted for infection history.
